# Anthocyanins: Molecular Aspects on Their Neuroprotective Activity

**DOI:** 10.3390/biom13111598

**Published:** 2023-10-31

**Authors:** César A. Zaa, Álvaro J. Marcelo, Zhiqiang An, José L. Medina-Franco, Marco A. Velasco-Velázquez

**Affiliations:** 1 School of Biological Sciences, Universidad Nacional Mayor de San Marcos, Lima 15021, Peru; 2 School of Biology, Universidad Nacional Federico Villarreal, Lima 15088, Peru; amarcelo@unfv.edu.pe; 3 Texas Therapeutic Institute, Brown Foundation Institute of Molecular Medicine, University of Texas Health Science Center, Houston, TX 77030, USA; zhiqiang.an@uth.tmc.edu; 4 DIFACQUIM Research Group, School of Chemistry, Universidad Nacional Autónoma de México, Mexico City CP 04510, Mexico; medinajl@unam.mx; 5 School of Medicine, Universidad Nacional Autónoma de México, Mexico City CP 04510, Mexico

**Keywords:** anthocyanins, antioxidants, food chemicals, natural products, neuroprotection, neurodegeneration

## Abstract

Anthocyanins are a type of flavonoids that give plants and fruits their vibrant colors. They are known for their potent antioxidant properties and have been linked to various health benefits. Upon consumption, anthocyanins are quickly absorbed and can penetrate the blood–brain barrier (BBB). Research based on population studies suggests that including anthocyanin-rich sources in the diet lower the risk of neurodegenerative diseases. Anthocyanins exhibit neuroprotective effects that could potentially alleviate symptoms associated with such diseases. In this review, we compiled and discussed a large body of evidence supporting the neuroprotective role of anthocyanins. Our examination encompasses human studies, animal models, and cell cultures. We delve into the connection between anthocyanin bioactivities and the mechanisms underlying neurodegeneration. Our findings highlight how anthocyanins’ antioxidant, anti-inflammatory, and anti-apoptotic properties contribute to their neuroprotective effects. These effects are particularly relevant to key signaling pathways implicated in the development of Alzheimer’s and Parkinson’s diseases. In conclusion, the outcome of this review suggests that integrating anthocyanin-rich foods into human diets could potentially serve as a therapeutic approach for neurological conditions, and we identify promising avenues for further exploration in this area.

## 1. Introduction

Polyphenols are natural compounds primarily found in fruits, vegetables, cereals, and natural juices. They serve as secondary metabolites in plants, aiding in defense against ultraviolet radiation and pathogens [1]. Polyphenols are abundant, often reaching up to 200–300 mg per 100 g of fresh weight. The amount of polyphenols in a glass of red wine or a cup of tea or coffee is approximately 100 mg [2]. The high levels of polyphenols in the human diet suggest their exceptional tolerance; however, the amount that can be safely and beneficially added for human consumption remains unclear [3]. Polyphenols are antioxidants and, therefore, may contribute to preventing diseases related to oxidative stress, such as cardiovascular and neurodegenerative diseases [4]. Additionally, they act as active agents in many medicinal plants, influencing various enzymes and cell receptors [5]. Beyond their antioxidant qualities, polyphenols represent a source of bioactive compounds that remain largely untapped in Western medicine [6].

Polyphenols are characterized chemically by their phenolic structures. These compounds exhibit diverse structures in the diet, ranging from simple molecules (monomers and oligomers) to polymers [7]. Most plant polyphenols exist as glycosides with different sugars and/or acylated sugars conjugated to their structures [8,9]. Polyphenols are classified based on their origin, biological function, and chemical structure [10]. For instance, among the over 8000 identified phenolic structures from natural sources, approximately 4000 are flavonoids. Flavonoids share a common structure consisting of two aromatic rings connected by an oxygenated heterocycle [11]. These compounds fall into six subclasses based on the heterocycle type: flavonols, flavones, isoflavones, flavanones, anthocyanidins, and flavanols (catechins and proanthocyanidins). Within each subclass, individual differences arise from varying numbers and arrangements of hydroxyl groups, along with degrees of alkylation and glycosylation [2].

Anthocyanins, a subset of flavonoids, are responsible for the red, purple, or blue colors in plants and fruits [12,13]. Over 600 anthocyanins have been identified in plants, demonstrating robust antioxidant capacity and a wide range of health benefits [14]. For example, in a meta-analysis of randomized controlled clinical trials, Park et al. (2021) showed that anthocyanin supplementation, up to 300 mg/day for four weeks, was sufficient to reduce body weight and body mass index [15]. In addition, a diet supplemented with anthocyanins reduces vascular and systemic inflammation in multiple clinical settings (reviewed by [16]), including type 2 diabetes patients [17].

Notably, it has been shown that anthocyanin consumption improves cognitive health. Dietary supplementation with blueberry anthocyanins improves cognitive performance in aging individuals [18], in elderly adults with cognitive impairment [19], in healthy adults [20,21,22,23], and in school children (7–10 years of age) [24]. Those effects correlate with improved brain perfusion and activation in brain areas associated with cognitive function and reduced cardiometabolic risk [25,26].

Furthermore, anthocyanins show significant potential in treating neurodegenerative diseases in various experimental models [27]. Given that neurodegenerative diseases share common pathogenic mechanisms, in the present narrative review, we aim to provide a detailed description of the effects of anthocyanins in the molecular pathways promoting neurodegeneration, such as neuroinflammation, oxidative stress, and excitotoxicity. Furthermore, we discuss the effects of anthocyanins on relevant in vitro and in vivo models and clinical studies. The evidence summarized here demonstrates that anthocyanins are compounds with multiple molecular targets and mechanisms of action that cooperate to elicit beneficial effects in patients with neurological conditions. 

## 2. Anthocyanins

### 2.1. Dietary Sources

Anthocyanins constitute the largest group of water-soluble pigments, contributing shades of pink, red, blue, or purple to the vacuolar sap of flowers and fruits’ epidermal tissues [28,29]. These compounds can also exist in colorless forms depending on pH levels. In their aglycone state (anthocyanidins), they exhibit instability. Although they resist light-induced degradation in plants, they are vulnerable to pH fluctuations and oxidative conditions. Glycosylation, often with glucose at the 3-position, and esterification with organic or phenolic acids prevent degradation. Additionally, complex formation with other flavonoids stabilizes anthocyanins [30].

**Figure 1:**
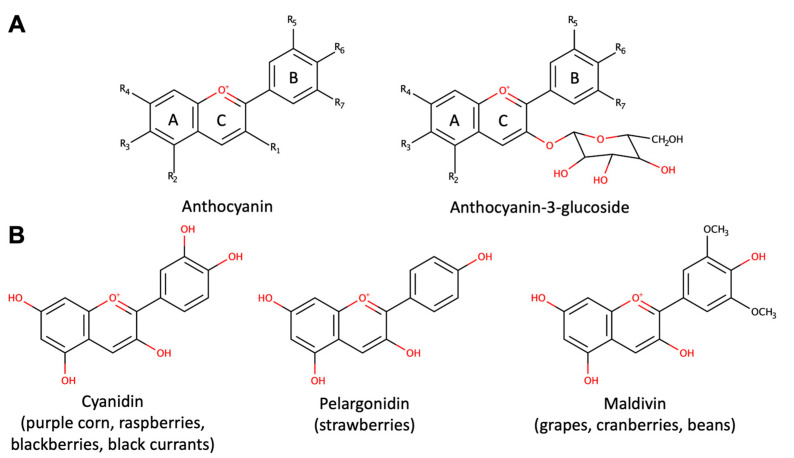
Structure of anthocyanins. (**A**) The general structures of anthocyanins and anthocyanins-3-glucoside. (**B**) Structures of bioactive anthocyanins with their common dietary sources.

Anthocyanins are present in red wine, some cereal varieties, and select leafy vegetables and roots (e.g., eggplants, cabbage, beans, onions, radishes). Red wine contains approximately 200–350 mg of anthocyanins per liter, with these compounds undergoing structural transformations as the wine matures [1]. However, fruits remain the primary source of anthocyanin consumption [31]. While mainly concentrated in the peel, certain red fruits, like cherries and strawberries, also contain anthocyanins in the pulp. Cyanidin (Figure 1B) stands out as the most prevalent anthocyanidin in foods [32]. Levels typically correlate with color intensity, with values reaching up to 2 to 4 g/kg of fresh weight in blackcurrants or blackberries, increasing as the fruits ripen [33]. 

### 2.2. Chemistry and Its Relationship with Bioavailability and Biodistribution

Anthocyanins exhibit a complex structure, characterized by glycosylation, polyhydroxy or polymethoxylated 2-phenylbenzopyryl derivatives, featuring two benzoyl rings (A and B) separated by a heterocyclic ring (C) (Figure 1A). The plant kingdom boasts around 400 distinct anthocyanins [29]. These flavonoids are distinguished by their heightened oxidation state, boasting a fully unsaturated C ring and a hydroxyl group at the 3-position [34]. Anthocyanins consist of an anthocyanidin (aglycone) bound to sugars (Figure 1A), often accompanied by organic acids in the case of acylated anthocyanins. Notably, variations in the number of sugars and binding positions, as well as the acylating groups of sugar substitutions, contribute to the diverse array of anthocyanin structures. Acylated anthocyanins exhibit pH-stable characteristics, tending to exhibit bluer hues compared to non-acylated counterparts. Favorable storage conditions involve cool, dark environments to maintain anthocyanin integrity due to the impact of light and temperature on anthocyanin degradation [34].

The therapeutic effects of anthocyanins require their absorption from dietary sources. Anthocyanins are susceptible to degradation in human saliva [35] and by the intestinal microbiota [36]. Due to their hydrophilicity, anthocyanins do not cross the gastrointestinal epithelium for paracellular absorption but use transporters. Anthocyanin glycosides can be translocated by sodium-dependent glucose transporter 1 (SGLT1), glucose transporter 2 (GLUT2), and organic anion-transporting polypeptide 2B1 (OATP 2B1), whereas the aglycon forms use only GLUT2 and OATP 2B1 [37]. After gastrointestinal absorption, anthocyanins are susceptible to phase II biotransformation, and their metabolites are transported in an ATP-dependent manner [38]. Because of all the above, some authors have expressed concerns that the therapeutic efficacy of anthocyanins may be hindered by their low bioavailability. 

Hahm and collaborators [37] recently summarized multiple in vivo pharmacokinetic studies with anthocyanins, finding that, in general, bioavailability is low. However, the physicochemical properties of each anthocyanin influence the bioavailability. For example, the presence of cationic groups within anthocyanin glycosides appears to render them resistant to enzymatic conjugation, facilitating efficient and rapid absorption as glycosides both in experimental animals and humans [39,40,41,42]. Anthocyanins like cyanidin-3-glucoside and cyanidin-3,5-diglucoside from fruits are incorporated into the liver and plasma of rats and humans, indicating that structurally intact glycoside forms of anthocyanins are efficiently absorbed from the digestive tract into the bloodstream [43,44]. In addition, the type of sugar bound to anthocyanins has been identified as an influential factor in their permeability and bioavailability [45]. 

Importantly, a growing body of evidence underscores the ability of anthocyanins to traverse the BBB [46,47]. Anthocyanins and their derivatives, such as cyanidin-3-rutinoside and pelargonidin-3-O-glucoside, are taken up by mouse and rat brain endothelial cells, revealing their capacity to penetrate the BBB [48]. Intravenous injection of anthocyanin cyanidin-3-O-b-D-glucoside further supports rapid brain uptake [49]. Andres-Lacueva et al. (2005) found anthocyanins like cyanidin-3-O-b-D-galactoside, cyanidin-3-O-b-D-glucoside, and cyanidin-3-O-b-D-arabinose in various brain regions of rats fed blueberry polyphenols, suggesting that dietary supplementation allows direct brain access [50]. Yet, the possible therapeutic use of anthocyanins in neurodegenerative diseases requires further research to identify strategies for improving bioavailability and biodistribution of anthocyanins with specific activities.

## 3. Mechanisms of Neuronal Damage Affected by Anthocyanins

Dementia, a shared hallmark of neurodegenerative diseases, stems from the targeted loss of specific neuronal cell populations within the central nervous system (CNS). Examples include the entorhinal cortex and hippocampus in Alzheimer’s disease (AD), the substantia nigra in Parkinson’s disease (PD), the striatum and cerebral cortex in Huntington’s disease (HD), and motor neurons in amyotrophic lateral sclerosis (ALS) [51]. While these diseases exhibit diverse underlying mechanisms, they share common features that intertwine [52]: (i) oxidative stress generation, (ii) inflammatory response, and (iii) excitotoxicity induction.

**Figure 2:**
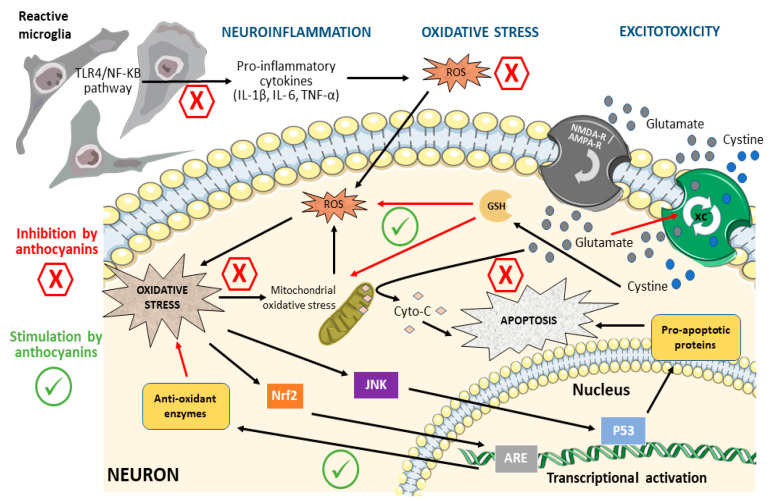
General scheme of the signaling pathways modulated by anthocyanins in neuroinflammation, oxidative stress, and excitotoxicity. In neuroinflammation, the activation of microglia (upper left) leads to the release of proinflammatory cytokines, promoting ROS generation. Exogenous or endogenous ROS can induce Oxidative stress in neurons, activating multiple pathways. Active Nrf2 promotes the expression of antioxidant enzymes by transactivating the antioxidant response element (ARE), whereas activation of the JNK/p53 pathway promotes apoptosis. Glutamate-driven excitotoxicity is promoted by an imbalance in the glutamate/cystine (XC-) antiporter system. An increased glutamate concentration induces the release of cytochrome-C (Cyto-C) and inhibits the entry of cystine, a key element for the formation of the antioxidant glutathione (GSH). GSH protects against ROS-mediated oxidative damage and mitochondrial dysregulation. Anthocyanins act in multiple stages of these pathways, decreasing proinflammatory signals, eliciting an antioxidant effect, or modulating the activation of proteins in the pathways (see text for details). Consequently, anthocyanins reduce neuroinflammation, oxidative stress, and/or apoptosis. Black arrows indicate direct or indirect stimulation, while red arrows represent inhibition or reduction. NMDA-R: NMDA receptor; AMPA-R: AMPA receptor; XC-: glutamate/cystine antiporter; Cyto-C: cytochrome-C.

Hence, targeting these shared features presents an appealing therapeutic avenue. Within this context, polyphenols have emerged as promising candidates thanks to their notable antioxidant, anti-neuroinflammatory, and anti-apoptotic properties [53]. Among them, anthocyanins hold considerable promise as potential treatments for neurodegenerative disorders [54]. These compounds, derived from diverse natural and dietary sources, have garnered significant attention and are undergoing investigation in various biological models to ascertain their neuroprotective potential by diverse mechanisms discussed below and summarized in Figure 2.

### 3.1. Oxidative Stress

Oxidative stress denotes an imbalance in cellular equilibrium resulting from cells’ inability to counteract the excessive production of free radicals [55]. The brain is particularly vulnerable due to its elevated lipid content, oxygen consumption, and relatively low antioxidant system activity. Consequently, the brain is more susceptible to oxidative stress than other organs [56]. 

Accumulation of reactive oxygen species (ROS) and reactive nitrogen species (RNS), combined with a dysfunction of the antioxidant system, leads to lipid, DNA, and protein damage in age-related neurodegenerative diseases like AD, PD and ALS [57,58,59]. For example, in models of AD, aggregation of beta-amyloid (bA) leads to mitochondrial dysfunction and oxidative stress prior to the development of plaque pathology [60]. Increased ROS production by bA is caused by the inhibition of mitochondrial complexes I and IV in neurons and astrocytes [61,62]. Similarly, mutations in the mitochondrial complex present in PD patients lead to the overproduction of superoxide [63]. Furthermore, oxidative stress perpetuates the release of proinflammatory molecules, exacerbating inflammation, which, in turn, is influenced by oxidative stress [64]. This cycle disrupts proper cell signal transduction regulation. Thus, oxidative stress in the brain compromises various neuronal functions, including synaptic plasticity, thus correlating with the emergence of neurodegenerative and psychiatric conditions [65,66]. Thus, reducing oxidative stress and modifying the release of mitochondrial ROS are desirable goals in therapeutic intervention of neurodegenerative diseases [67].

A recent systematic review of randomized controlled trials in humans showed a significant effect of consuming berries on biomarkers related to oxidative stress [68]. Anthocyanins’ neuroprotective effects are closely tied to their antioxidant and radical scavenging abilities. Various forms of anthocyanins, including pure compounds, anthocyanin-enriched fractions, and their metabolites, shield neuronal model cells from hydrogen peroxide-induced cell death [14,69,70], ethanol-induced damage [71], oxygen–glucose deprivation [72], and bA peptide toxicity [73] by mitigating oxidative stress.

Moreover, anthocyanins exhibit protective effects against mitochondrial oxidative stress (MOS). Cerebellar granule neuron cultures hamper MOS-induced apoptosis by preserving mitochondrial glutathione (GSH) levels, inhibiting cardiolipin oxidation, and preventing mitochondrial fragmentation. These actions collectively contribute to substantial protection against induced apoptosis [74].

In mammalian cells, the main regulator of the antioxidant response is the Nrf2/antioxidant response element (ARE) pathway [75]. Nrf2 is a transcription factor that binds to ARE, coordinating the expression of multiple antioxidant enzymes, including glutamate cysteine ligase (GCL), thioredoxin reductase 1 (Txnrd1), NAD(P)H-quinone oxidoreductase 1 (NQO1) and heme oxygenase-1 (HMOX1) [76,77]. The protective activities of anthocyanins are partially caused by their ability to induce Nrf-2 activation [78,79]. The administration of a diet rich in anthocyanins to aged rats significantly elevates Nrf2 levels in the hippocampus and prefrontal cortex as well as the expression of antioxidant enzymes such as superoxide dismutase 1 (SOD1) and glutathione S-transferase [80]. Similar effects have been reported in vitro. The berries’ anthocyanin cyanidin-3-glucoside [81] and commercial proanthocyanidins [82] induce the Nrf2 antioxidant defense system, reducing oxidative stress and apoptosis in stressed cultured neurons. This evidence suggests a key role of Nrf2 activation in the neuroprotective effects of anthocyanins and calls for the identification of additional Nrf2 activators for controlling oxidative stress in the brain. 

### 3.2. Excitotoxicity 

Excitotoxicity denotes cell demise triggered by the actions of excitatory amino acids within the nervous system. Neurons susceptible to excitotoxicity play pivotal roles in functions such as learning and memory. Given that the neurotransmitter glutamate is a primary excitatory neurotransmitter in the mammalian CNS, neuronal excitotoxicity largely results from prolonged exposure to glutamate [83]. A key pathogenic mechanism of excitotoxicity is oxytosis [84]. Oxycytosis is an oxidative stress-induced cell death pathway [85,86] that can be induced by extracellular glutamate. Elevated brain glutamate levels lead to neurotoxicity, triggering heightened intracellular ROS [83,87]. Increased extracellular glutamate concentrations hinder cystine influx into cells, diminishing intracellular glutathione antioxidant levels and inducing oxidative stress [88]. Moreover, glutathione depletion accelerates multiple downstream signaling pathways, ultimately leading to neuronal demise via abnormal calcium uptake and lipid peroxidation [89]. This calcium overload activates catabolic enzymes that degrade proteins, membranes, and nucleic acids, culminating in neurotoxicity [90].

Numerous in vitro studies underscore that at elevated concentrations, glutamate is a potent neurotoxin capable of eliciting neuronal apoptosis [91]. Glutamate-triggered cell death in primary cortical neurons and HT22 neurons involves positive regulation of a caspase-dependent pathway with mitochondrial signaling involvement [92]. Those effects are primarily attributed to the glutamate/cystine antiporter imbalance and its repercussions on cytosolic homeostasis [93]. Kainic acid, a non-degradable glutamate analog, is utilized in brain injury models and induces cell death in HT22 neurons and primary hippocampal neurons [94,95,96]. The cytotoxicity of kainic acid correlates with increased ROS levels and caspase-3 activation. Pretreatment with anthocyanins from the “Korean black soybean” *Glycine max* (L.) Merr. (*Fabaceae*), at 100 and 200 µg/mL, significanltly mitigates kainic acid-induced cell viability loss [95]. Similarly, anthocyanin-containing extracts from *Vitis vinifera* L. grapes [97] and black capulin cherry tree [98] significantly reduced glutamate-triggered cell death in HT22 cells. Notably, anthocyanins’ in vivo neuroprotective effect was demonstrated by Shah et al. (2016), revealing that anthocyanins can alleviate glutamate-induced neurotoxicity in the developing rat brain through an AMPK-dependent mechanism [99]. Purified cyanidin-3-O-galactoside [98] mirrors the neuroprotective impact of whole extracts by mitigating glutamate-induced oxidative stress, preserving mitochondrial function, and reducing caspase-dependent apoptosis. A similar effect has been reported for astaxanthin [100], which is commercialized in multiple countries as a supplement, underscoring the potential of anthocyanins isolated from natural sources for further investigation.

### 3.3. Neuroinflammation 

Neuroinflammation is a feature of neurodegenerative diseases. Persistent inflammation in the brain affects neural plasticity, impairs memory, and is considered a typical driver of neurodegenerative disorders [101]. Multiple cells are located within the CNS, including neurons, macroglia, and microglia. Microglia constitute the most abundant resident macrophages in the CNS, with roles in tissue defense and repair [102]. Dysregulated activation of microglia and astrocytes within the CNS and infiltrating immune cells has been observed in various neurodegenerative conditions [103,104]. The accumulation of inflammatory cells and soluble mediators leads to sustained neuroinflammation and sensitizes neurons to further insults, triggering neurodegeneration [104]. Accordingly, therapies in neurodegenerative diseases aim to reduce the activation of astrocytes and microglia, and the concentration of proinflammatory cytokines [67].

Various factors such as environmental, genetic, and age merge to activate microglia and trigger inflammatory pathways [105]. For example, lipopolysaccharide binding to Toll-like receptor 4 (TLR4) on the surface of microglia activates phosphoinositide 3-kinase/protein kinase B (PI3K/AKT), mitogen-activated protein kinase (MAPK) and mammalian target of rapamycin (mTOR) pathways, which ultimately lead to NF-κB activation [106,107]. In turn, activation of NF-κB mediates the production of proinflammatory molecules, including iNOS, interleukin 1 beta (IL-1β), tumor necrosis factor-alpha (TNF-α), cyclooxygenase-1 (COX-1), COX-2, and ROS, resulting in neuroinflammation [108] and potentially in neuronal dysfunction and cell death [109,110]. 

Poulose et al. (2012) showed that anthocyanins from the pulp of the açai fruit (*Euterpe oleracea* Mart.) attenuate inflammatory stress signaling in mouse brain BV-2 microglial cells through the reduction of COX- 2, p38-MAPK) TNFα, and NF-κB [111]. Anthocyanins from black soybean seed coats significantly inhibited proinflammatory mediators induced by lipopolysaccharide in BV2 microglial cells, such as nitric oxide (NO) and prostaglandin E 2, and proinflammatory cytokines such as TNF-α and IL-1β. They also inhibited the nuclear translocation of NF-κB by reducing the degradation of the inhibitor IκB-alpha and the activation of the AKT/JNK pathway [112].

Neuroinflammation reduction by anthocyanins may impact the progression of neurodegenerative diseases. For example, anthocyanins (alone and loaded in nanoparticles) decrease the expression of neuroinflammatory and apoptotic markers in Aβ (amyloid beta peptide) 1-42-injected mouse models of AD and in vitro by inhibiting the p-JNK/NF-κB/p-GSK3β pathway [113]. In a multiple sclerosis (MS) model, neuronal homeostasis is restored by the administration of total anthocyanins from the grape (*Vitis vinifera*) skin. Treatment reduces the expression of proinflammatory cytokines IL-1β and TNF-α and increases the expression of anti-inflammatory cytokines (i.e., IL-10), reducing inflammatory cell infiltration [114]. 

### 3.4. Altered Cholinergic Transmission

Reduction in cholinergic transmission, caused by alterations in acetylcholine (ACh) synthesis and release and decreased expression of cholinergic receptors, contribute to the cognitive impairment observed in patients with AD [115]. Acetylcholinesterase (AChE) catalyzes the hydrolysis of ACh to choline and acetate, terminating ACh-mediated synaptic transmission. Thus, an approach to treat AD is the use of AChE inhibitors [115], which reduce the breakdown of endogenously released ACh, increasing ACh levels in the synaptic cleft and amplifying the activation of postsynaptic cholinergic receptors [116]. 

Anthocyanins from grape skin [117] or passion fruit epicarp extract [118] inhibit AChE with IC_50_ of 363.61 µg/mL and 18.29 mg/mL, respectively. Similarly, the peel of hawthorn fruit (*Crataegus pinnatifida* Bge. var. *major*) [119] and Morus fruit extract [120], mainly containing anthocyanins/anthocyanidins, inhibit AChE. Red-leaf tea extract, which is rich in delphinidin and cyanidin-3-O-galactosides, inhibits AChE [121]. Structure–activity relationship studies have shown that the hydroxyl groups in the three anthocyanin rings are important for the AChE inhibitory activity. Thus, glycosylation in positions 3 (C ring) or 5 (A ring) decreases their potency, whereas the presence of hydroxyls in 3’ and 5’ (B ring) promotes AChE inhibition [122]. Accordingly, anthocyanins have become lead compounds for the development of more effective AChE inhibitors [123]. 

## 4. Effect of Anthocyanins on Neurological Disorders

Due to their ability to modulate mechanisms implicated in the onset of neurological diseases, anthocyanins hold significant potential for treating such conditions [27]. In this section, we focus on three prevalent diseases with limited therapeutic options: AD, PD, and brain ischemia.

### 4.1. Alzheimer’s Disease

The protective impact of anthocyanins against AD is underscored by their potential to delay disease progression. Consistent with this, studies reveal that consistent consumption of fruits, vegetables, and beverages like green tea and red wine (in moderation) diminishes the risk of age-related neurological disorders, including AD [124,125]. Examination of data on AD prevalence and incidence in relation to genetic and environmental factors suggests that the use of antioxidant supplements correlates with reduced occurrence of AD [126].

In animal models, anthocyanins have demonstrated AD-delaying effects. For instance, in a mutant AD mouse model, anthocyanins from blueberry and black currant impeded Aβ deposition and mitigated cognitive impairment [127]. Cranberry anthocyanins avert memory and learning deficits in rats induced by streptozotocin injection by regulating ion pumps and cholinergic neurotransmission [128]. Anthocyanins in gold nanoparticles alleviate memory loss and neurodegeneration in mice with Alzheimer’s-like symptoms by reducing Aβ, beta-secretase (BACE-1), and amyloid precursor protein levels [113]. Administration of cyanidin-3-glucoside orally halted cognitive decline induced by Aβ peptide [129].

Mechanistically, the effects of anthocyanins in AD mouse models relate to changes in Aβ deposition. Anthocyanin mixtures hinder Aβ oligomerization and subsequent tau phosphorylation, potentially curbing tau protein aggregate formation [130]. Cranberry-derived anthocyanin-rich extracts hinder Aβ1-40 and Aβ1-42 peptide formation in vitro, diverting these peptides to non-toxic aggregates. These interventions preserve cognitive function in disease-model mice [131]. Cyanidin-3-O-glucopyranoside anthocyanins [132] and malvidin (Figure 1B) [131] directly interfere with Aβ peptide oligomerization into toxic fibrils. These effects may be tied to microglia activation. Blueberry anthocyanins enhance microglial Aβ peptide clearance, inhibiting aggregation via the mitogen-activated protein kinases (MAPKs) pathway [133]. 

An enhanced antioxidant response is also elicited by anthocyanins. In a model of sporadic Alzheimer’s dementia induced by streptozotocin, commercial anthocyanins from grape skins decreased lipid peroxidation. They restored the level of antioxidant enzymes, such as superoxide dismutase (SOD), catalase (CAT), and glutathione peroxidase (GPx), in the cortex and hippocampus [134]. Anthocyanins from black soybean (*Glycine max* (L.) Merr.) also exhibit direct protection against neuronal cytotoxicity induced by bA injected in the hippocampus [135]. Black soybean anthocyanins enhance HT22 neuron cell viability compared to Aβ-treated cells [135], and blueberry anthocyanins prevent ROS formation and cognitive decline [127]. Natural anthocyanins from Korean black beans reduce ROS levels in mice with high Aβ production and HT22 cells exposed to Aβ oligomers [113,136]. In summary, anthocyanins present promise for AD treatment and prevention, potentially supplementing current therapies due to their established safety. 

Finally, the effect of anthocyanins on AD is associated with the regulation of cholinergic neurotransmission by AChE inhibition. In vivo studies showed that the administration of anthocyanins protects against the increase of AChE in the cortex hippocampus [134,137] and cerebellum [138] in models of cognitive deficits associated with AD. In addition, administration of anthocyanin-rich blueberry extract (*Vaccinium angustifolium*) to mice decreases AChE activity [139]. The inhibition was significantly higher in the brain compared to other tissues [139], suggesting a preferential biodistribution or a selective binding to the AChE isoform expressed in the brain. Treatment of mice with spatial memory impairment with delphidin (50 mg/Kg) reduced AChE activity and amyloid plaque formation in the brain [140]. 

### 4.2. Parkinson’s Disease (PD)

Epidemiological evidence suggests that consumption of anthocyanin-rich berries, like blueberries or strawberries, may mitigate the risk of PD [141]. Clinical studies indicate that blackcurrant (*Ribes nigrum*) anthocyanins augment the neuroprotective cyclic glycine-proline concentration in Parkinson’s patients’ cerebrospinal fluid [142]. An anthocyanin-rich blackberry extract showed preventive effects against bradykinesia and dopaminergic neuronal damage induced by 1-methyl-4-phenyl-1,2,3,6-tetrahydropyridine (MPTP) in a PD model [143].

In PD, dopaminergic cell death involves mitochondrial complex I impairment, oxidative stress, microglial activation, and Lewy body formation. Blueberry and grape seed extracts restored mitochondrial respiration defects caused by rotenone exposure in dopaminergic cell lines, suggesting improved mitochondrial function and potential neurodegeneration alleviation [144]. Anthocyanins from grape (*Vitis vinifera*) and Japanese knotweed (*Polygonum cuspidatum*) enhanced climbing ability in a transgenic Drosophila PD model expressing human alpha-synuclein [145]. Pelargonidin (Figure 1B) anthocyanin exhibited neuroprotective effects against 6-OHDA-induced toxicity by reducing oxidative stress [146]. Anthocyanins from mulberry (*Morus alba* L.) fruit protected dopaminergic neurons against MPTP exposure by regulating ROS and NO generation, reducing Bcl-2 and Bax expression, mitochondrial membrane depolarization, and caspase-3 activation [143]. 

### 4.3. Hypoxia/Cerebral Ischemia

Ischemic stroke is due to a transient or permanent reduction in cerebral blood flow. The main mechanisms of ischemia/reperfusion injury include excitotoxicity, oxidative stress, inflammation, and apoptosis [147]. In murine models of cerebral ischemia/reperfusion injury, treatment with commercial anthocyanins [148], anthocyanins obtained from the dried fruits of *Lycium ruthenicum* Murr. [149], or purified anthocyanins from *Myrica rubra* [150] protect against middle cerebral artery occlusion injury, altering apoptosis and inflammation. Similarly, cyanidin 3-O-β-glucopyranoside has a neuroprotective effect in an animal cerebral artery occlusion model [151]. In mice under conditions of transient global ischemia, anthocyanins from black rice (*Oryza sativa* L., Poaceae) attenuate neuronal cell death, inhibit reactive astrogliosis, and prevent loss of expression of glutathione peroxidase in the hippocampus, significantly improving memory impairment [152].

As for other diseases discussed above, the amelioration of neuronal injury by anthocyanins in ischemia/reperfusion can be partially explained by their anti-apoptotic, anti-inflammatory, and anti-oxidative activities. Anthocyanins reduce neuronal apoptosis induced by ischemia and/or reperfusion through regulation of the expression of Bcl-2 family proteins [149], reduction of cytochrome c and caspase-3 [153,154,155], and suppression of JNK/p53 pathway [156]. Purified extracts of *Myrica rubra* anthocyanins protect against cerebral ischemia-reperfusion injury by modulating the TLR4/NF-κB, NLRP3, and Nrf2 signaling pathways [150] and by reducing the levels of inflammatory molecules, including TNF-α, IL-1β, and IL-6 [149]. In neuron cultures, anthocyanins from black soybean (*Glycine max* (L.) or purified cyanidin-3-glucoside protect from the cytotoxicity induced by oxygen–glucose deprivation by inhibiting oxidative stress and preserving the mitochondrial membrane potential [157]. The effects and mechanism of anthocyanins described here make them candidates for consideration as a dietary supplement to reduce ischemia injury. 

## 5. Conclusions

Anthocyanins are natural compounds with good safety profiles and biodistribution to the CNS. Evidence from in vitro studies and animal models supports the beneficial actions of dietary anthocyanins on neurodegenerative diseases, such as AD and PD, and ischemic brain damage. Since oxidative stress, inflammation, cytotoxicity, and altered neurotransmission are common mechanisms underlying the etiology of those diseases, the neuroprotective effects of anthocyanins have been explained by (Figure 2): (i) their ability to eliminate pathological concentrations of ROS and NRS either by acting directly as radical scavengers or by promoting the expression of antioxidant enzymes; (ii) the reduction of activation of inflammatory pathways in SNC; (iii) their cytoprotective and anti-apoptotic effects on neurons; and (iv) the promotion of cholinergic neurotransmission. Eliciting such mechanisms, anthocyanins reduce cellular damage and improve cognitive function. Together, the findings discussed here underscore the potential utility of anthocyanins in neuroprotection. However, there are still areas of opportunity in the field. First, the evidence from clinical studies is still limited, and thus, future research should explore the efficacy of anthocyanins or anthocyanin-rich supplements in specific human diseases using relevant populations. In those studies, the pharmacokinetic and pharmacodynamic interactions between anthocyanins and standard-of-care drugs should be ruled out. Second, structure–activity relationships should be established to identify the new candidates for each potential clinical translation. Those analyses could also allow the design of new anthocyanin-like molecules with selective or enhanced activities.

## Abbreviations

6-OHDA: 6-hydroxydopamine; acetylcholine: ACh; Acetylcholinesterase: AChE; Alzheimer’s disease: AD; amyotrophic lateral sclerosis: ALS; amyloid precursor protein: APP; antioxidant response element: ARE; beta-amyloid: bA; blood–brain barrier: BBB; Catalase: CAT; central nervous system: CNS; cyclooxygenase-1: COX-1; glucose transporter 2: GLUT2; glutamate-cysteine ligase: GCL; glutathione: GSH; glutathione peroxidase: GPx; heme oxygenase-1: HMOX1; Huntington’s disease: HD; interleukin 1 beta: IL-1β; lipopolysaccharide: LPS; mammalian target of rapamycin: mTOR; mitochondrial oxidative stress: MOS; mitogen-activated protein kinase: MAPK; 1-methyl-4-phenyl-1,2,3,6-tetrahydropyridine: MPTP; multiple sclerosis: MS; NAD(P)H-quinone oxidoreductase 1: NQO1; nitric oxide: NO; organic anion-transporting polypeptide 2B1: OATP 2B1; Parkinson’s disease: PD; phosphoinositide 3-kinase/protein kinase B: PI3K/AKT; reactive nitrogen species: RNS; reactive oxygen species: ROS; sodium-dependent glucose transporter 1: SGLT1; superoxide dismutase: SOD; thioredoxin reductase 1: Txnrd1; Toll-like receptor 4: TLR4; tumor necrosis factor alpha: TNF-α; Aβ: amyloid beta peptide.
